# Identification and Characterization of MAPK Signaling Pathway Genes and Associated lncRNAs in the Ileum of Piglets Infected by *Clostridium perfringens* Type C

**DOI:** 10.1155/2020/8496872

**Published:** 2020-08-12

**Authors:** Ruirui Luo, Xiaoyu Huang, Zunqiang Yan, Xiaoli Gao, Pengfei Wang, Qiaoli Yang, Wei Wang, Kaihui Xie, Shuangbao Gun

**Affiliations:** ^1^College of Animal Science and Technology, Gansu Agricultural University, Lanzhou 730070, China; ^2^Gansu Research Center for Swine Production Engineering and Technology, Lanzhou 730070, China

## Abstract

*Clostridium perfringens* type C (*C. perfringens* type C) is one of the main microbial pathogens responsible for piglet diarrhea worldwide, causing substantial economic losses for pig-rearing industries. The mitogen-activated protein kinase (MAPK) signaling pathway is a key regulator of inflammatory bowel disease, especially necrotic enteritis. However, whether and how the MAPK signaling pathway is involved in regulating the process of piglet diarrhea when challenged by *C. perfringens* type C are still unknown. Here, we screened 38 differentially expressed genes (DEGs) in piglets' ileum tissues experimentally infected with *C. perfringens* type C that were enriched in the *Sus scrofa* MAPK signaling pathway, based on our previous transcriptome data. Of these DEGs, 12 genes (*TRAF2*, *MAPK8*, and *GADD45G*, among others) were upregulated whereas 26 genes (*MAPK1*, *TP53*, and *CHUK*, among others) were downregulated in the infected group. Our results showed that MAPK1, TP53, MAPK8, MYC, and CHUK were in the core nodes of the PPI network. Additionally, we obtained 35 lncRNAs from the sequencing data, which could be *trans*-targeted to MAPK signaling pathway genes and were differentially expressed in the ileum tissues infected with *C. perfringens*. We used qRT-PCR to verify the expression levels of genes and lncRNAs related to the MAPK signaling pathway; their expression patterns were consistent with RNA sequencing data. Our results provide strong support for deeply exploring the role of the MAPK signaling pathway in diarrhea caused by *C. perfringens* type C.

## 1. Introduction

Piglet diarrhea is one of the most severe diseases afflicting piglets, leading to their delayed growth and development, low feed returns, and even death, which has seriously damaged the economic development of pig industries globally [1]. Recently, *Clostridium perfringens*, the important pathogenic microorganism that causes diarrhea in piglets, was divided into five toxinotypes: A, B, C, D, and E [2]. The *Clostridium perfringens* type C (*C. perfringens* type C) is a gas-tolerant bacterium widely distributed in nature that may cause various diseases in animals, including cellulitis, gas gangrene, intestinal toxemia, and necrotic enteritis [2, 3]. Importantly, *C. perfringens* type C can produce alpha and beta toxins, which are known to play critical roles in intestinal epithelial cell damage and necrosis, as well as intestinal inflammatory responses [4, 5].

The mitogen-activated protein kinase (MAPK) signaling pathway is known to participate in various biological processes including innate immunity, cell growth, stress response, apoptosis, and differentiation [6]. The mammalian MAPK family includes three subfamilies, namely, extracellular signal-regulated kinases (ERKs), c-Jun N-terminal kinases (JNKs), and p38 MAPKs [7]. The MAPK signaling pathway is one of the major pathways activated by cells following infection and intoxication [8]. The *C. perfringens* alpha toxin can induce the release of cytokine *IL-8* by activating the ERK1/2 and p38 MAPK signaling pathways [9], while the *C. perfringens* beta toxin can cause the phosphorylation of p38 and JNK [10]. It has been reported that p38, JNK1/2, and ERK1/2 may be activated in the course of inflammatory bowel disease (IBD) [11–13].

Long noncoding RNAs (lncRNAs) are a type of noncoding RNA molecules longer than 200 nucleotides, which play an important role in many physiological and pathological processes [14, 15]. lncRNA H19 can promote the development of bronchopulmonary dysplasia by regulating the MAPK signaling pathway, and the MAPK signaling pathway can be used as a potential target for the treatment of bronchopulmonary dysplasia [16]. Jiang et al. found that lncRNA MALAT1 can promote high glucose-induced apoptosis of rat cartilage endplate cells through the p38/MAPK signaling pathway [17]. At present, studies have confirmed that lncRNA H19 [18], lncRNA NEAT1 [19], and lncRNA BC012900 [20] play an important role in IBD by regulating the intestinal epithelial barrier. Identifying lncRNAs related to MAPK signaling pathway genes is very necessary to study piglet diarrhea caused by *C. perfringens* type C.

Currently, there are no published literature reports on differential expression and regulation of genes related to the MAPK signaling pathway in diarrhea piglets caused by *C. perfringens* type C. In our preliminary transcriptome study, we have identified 25491 mRNAs and 3740 lncRNAs in the ileum tissues of piglets infected with *C. perfringens* type C [21]. Building on this, the present work was designed to further investigate the expression patterns of MAPK signaling pathway genes in the ileum tissues of infected piglets using quantitative real-time polymerase chain reaction (qRT-PCR). In addition, we screened differentially expressed lncRNAs related to the MAPK signaling pathway based on an integrated analysis of lncRNAs and mRNAs. Collectively, these results will reveal the expression patterns of the MAPK signaling pathway genes in the diarrhea-stricken ileum of piglets infected with *C. perfringens* type C, which provides a valuable basis for further breeding of diarrhea-resistant piglet strains.

## 2. Materials and Methods

### 2.1. Ethics Statement

All experimental procedures using animals were performed in accordance with the regulations for the Administration of Affairs Concerning Experimental Animals (Ministry of Science and Technology, China; revised in June 2004). This study was approved by the ethics committee of the College of Animal Science and Technology, Gansu Agricultural University (approval number 2006-398). All efforts were taken to minimize suffering in the animal subjects.

### 2.2. The *C. perfringens* Type C Culture, Animal Treatment, and Sample Collection

Thirty 7-day-old suckling piglets (Yorkshire sow×Landrace boar) from Dingxi city in Gansu Province, China, were selected as the experimental subjects. These piglets were not infected with *Escherichia coli*, *Salmonella*, or *C. perfringens* as determined by commercial enzyme-linked immunosorbent assay (ELISA) kits (Jiancheng Bioengineering Institute, Nanjing, China). Twenty-five experimental pigs were randomly selected to serve as the infected group, while the remaining five formed the control group (IC). The *C. perfringens* type C strain (CVCC 2032) was purchased from the China Veterinary Culture Collection Center (Beijing, China). Bacteria were cultured using the methods described in Huang et al. [22]. Each piglet was fed 1 mL of the *C. perfringens* type C culture medium (1 × 10^9^ CFU/mL) daily for 5 days. Fecal symptoms were monitored and recorded daily during the infection period; using a previously described method [23, 24], they were judged and scored as follows: 0: normal, solid feces; 1: slight diarrhea, soft and loose feces; 2: moderate diarrhea, semiliquid feces; and 3: severe diarrhea, liquid and unformed feces. According to the summed diarrhea scores, 25 piglets were ranked from high to low. The top five piglets and the bottom five were designated as the susceptibility (IS) and resistance (IR) groups, respectively. The ileum tissues from the IR, IS, and IC groups were collected and flushed cleanly with a PBS buffer (pH 7.4), then quickly frozen in liquid nitrogen and stored at -80°C until RNA extractions.

### 2.3. RNA Extraction and High-Throughput RNA Sequencing

The total RNA was extracted from ileum tissues using the TRIzol reagent (Invitrogen, Carlsbad, CA, USA). The purity of RNA samples was assessed using a NanoPhotometer spectrophotometer (Implen, Westlake Village, CA, USA). Ileum total RNA quantity and integrity were measured using a Qubit 2.0 Fluorometer (Life Technologies, Carlsbad, CA, USA) and RNA 6000 Nano Assay Kit of the Bioanalyzer 2100 system (Agilent Technologies, Santa Clara, CA, USA), respectively. Approximately 3 *μ*g rRNA-depleted RNA (Ribo-Zero RNA) was acquired from total RNA by an Epicentre Ribo-Zero™ rRNA Removal Kit (Epicentre, USA) and cleaned up by ethanol precipitation. The cDNA libraries were constructed and sequenced on a HiSeq 4000 platform (Illumina, San Diego, CA, USA).

### 2.4. Identification of Differentially Expressed Genes (DEGs) and Differentially Expressed lncRNAs

All of the raw sequencing data were deposited into a sequence read archive (SRA), under accession number PRJNA399620, at the National Center for Biotechnology Information (NCBI). Based on previous gene expression profiles obtained from RNA-seq [21], a total of 25491 mRNAs were identified in the ileum tissues of piglets. Among these, we screened genes with fold change of ≥1.5, *p* value < 0.05, and FPKM value > 1 from IR vs. IC and IS vs. IC as differentially expressed genes. We selected the 2004 DEGs from IR vs. IC for subsequent analysis (Table S1-1). In the sequencing data, we screened the lncRNAs of differentially expressed MAPK signaling pathway genes by *trans* and then selected the differentially expressed lncRNAs with *p* value < 0.05 as standard.

### 2.5. Pathway and Clustering Analyses of DEGs

Pathway enrichment analysis for DEGs was performed with KEGG database using DAVID online software (https://david.ncifcrf.gov/) [25]. We used Fisher's exact test to screen out significant enrichment pathways related with immunity (*p* < 0.05). The genes detected in a candidate immune system-related pathway (*Sus scrofa* MAPK signaling pathway) were subjected to hierarchical clustering, using the OmicShare tools (http://www.omicshare.com/tools).

### 2.6. Construction of Protein-Protein Interaction (PPI) Networks of Genes Associated with the MAPK Signaling Pathway

To assess the interactions among genes associated with the MAPK signaling pathway, the PPI network of proteins coded by the obtained DEGs was built, by using the “Search Tool” for the “Retrieval of Interacting Genes/Proteins” (STRING) database (https://string-db.org/) [26]. In the STRING database, we chose *Sus scrofa* as the organism while setting the edge of the network as confidence. We chose textmining, experiments, databases, coexpression, neighborhood, gene fusion, and cooccurrence as the active interaction source and chose a medium confidence level (0.400). The thickness of the line connecting any two genes indicates the strength of the data support.

### 2.7. Expression Levels of lncRNAs and Genes Associated with the MAPK Signaling Pathway

Based on the results above, 25 genes and 5 lncRNAs associated with the MAPK signaling pathway were randomly selected for further quantitative determination by qRT-PCR. The RNA samples used for qRT-PCR were derived from the samples used for sequencing. One microliter of total RNA (500 ng/*μ*L) was reverse-transcribed into cDNA using a PrimeScript™ RT Reagent kit (TaKaRa, Dalian, China). Primers were designed for each gene using the BLAST online software provided by the NCBI database and then synthesized by GENEWIZ Co. Ltd. (Tianjin, China) (Table S2). The qRT-PCR was performed on a LightCycler 480 II platform (Roche, Basel, Switzerland). A final volume of 20 *μ*L for the qRT-PCR reaction system consisted of 10 *μ*L of 2x SYBR Green Real-time PCR Master Mix (TaKaRa, Dalian, China), 0.8 *μ*L of forward and reverse primers (10 *μ*mol), 2 *μ*L of cDNA (500 ng/*μ*L), and 6.4 *μ*L of RNase-free ddH_2_O. The cycling conditions included an initial activation phase at 95°C for 3 min, followed by 40 cycles at 95°C for 15 s (denaturation) and at 60° ± 1°C for 15 s (annealing), with an extension phase at 72°C for 20 s. The mRNA and lncRNA abundances were calculated using the 2^−*ΔΔ*Ct^ method [27]. Three technical replicates were performed for each sample.

### 2.8. Statistical Analysis

All qRT-PCR experimental data were analyzed using one-way analysis of variance. Statistical significance was determined using the two-tailed Student's *t*-test method. The results are expressed here as mean ± SD (standard deviation). A *p* value < 0.05 and fold change > 2 were considered statistically significant; a *p* value < 0.01 and fold change > 2 were interpreted as highly significant.

## 3. Results

### 3.1. Acquisition of DEGs and Screening of MAPK Signaling Pathway-Related Genes

**Figure 1:**
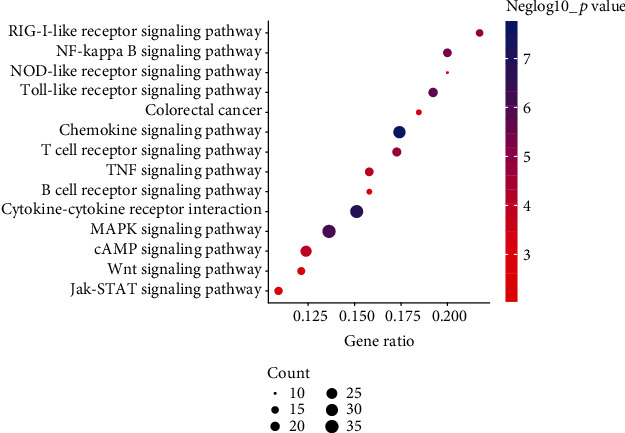
The bubble plot showing the immune-related KEGG signaling pathways significantly enriched by DEGs. All DEGs in the ileum tissue of *C. perfringens* type C-induced piglets were subjected to a comparative KEGG database search to identify their involvement in immune system-related pathways. The gene ratio is on the *x*-axis and the KEGG pathway names are on the *y*-axis. A dot's size is proportional to the number of target genes, and its coloring indicates differing negLog_10_*p* values.

**Figure 2:**
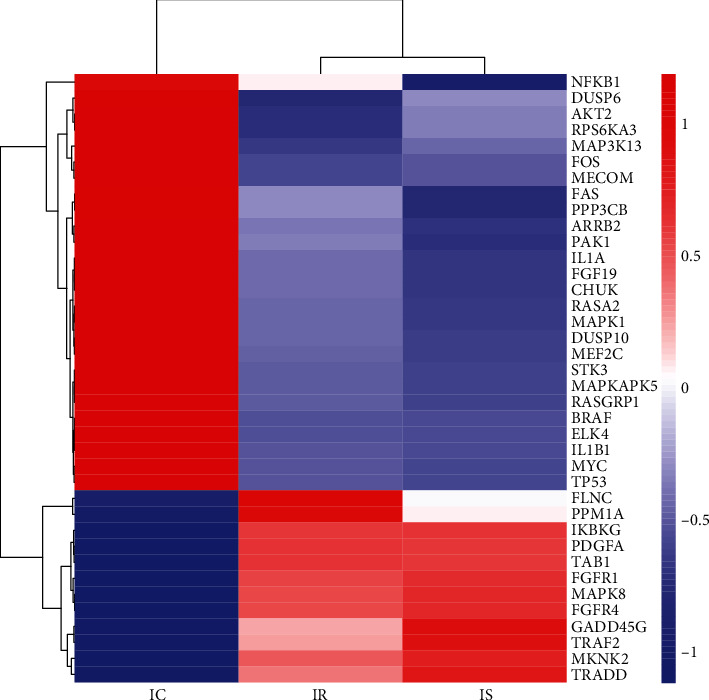
Clustering of genes involved in the MAPK signaling pathway. Hierarchical clustering of 38 DEGs in the ileum tissue of *C. perfringens* type C-induced piglets (IR and IS) relative to the control group (IC) that are involved in the MAPK signaling pathway. Upregulated and downregulated genes relative to the mean are, respectively, colored red and blue. Rows represent the mRNAs while columns represent different treated groups.

Based on the results of the KEGG analysis, we selected the first 14 types of significant enrichment pathways related to the immune system (Table S1-2), such as the MAPK signaling pathway, NF-kappa B signaling pathway, T cell receptor signaling pathway, nucleotide-binding oligomerization domain-containing protein- (NOD-) like receptor signaling pathway, retinoic acid-inducible gene 1 protein- (RIG-I-) like receptor signaling pathway, and toll-like receptor signaling pathway (Figure 1). Furthermore, a total of 38 DEGs from the infected piglet groups (IR and IS), consisting of 12 upregulated and 26 downregulated genes, were involved in the MAPK signaling pathway when compared with the IC group (Table 1). Hierarchical clustering of the 38 DEGs in ileum tissues from IR, IS, and IC showed hat the two infected groups were clustered (Figure 2). Among these DEGs, TNF receptor-associated factor 2 (*TRAF2*), mitogen-activated protein kinase 8 (*MAPK8*), fibroblast growth factor receptor 1 (*FGFR1*), and growth arrest and DNA damage inducible gamma (*GADD45G*) were all upregulated in the IR and IS groups, whereas the conserved helix-loop-helix ubiquitous kinase (*CHUK*), mitogen-activated protein kinase 1 (*MAPK1*), AP-1 transcription factor subunit (*FOS*), and tumor protein P53 (*TP53*) genes were downregulated (Figure 2).

### 3.2. Distribution Positions and PPI Network of DEGs Located in the MAPK Signaling Pathway

**Figure 3:**
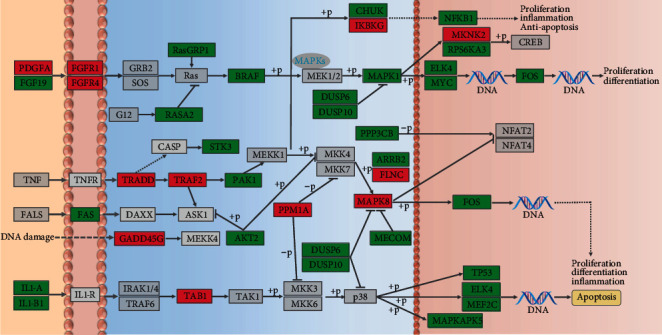
Localization of 38 DEGs in the MAPK signaling pathway. These respective positions were marked in the *Sus scrofa* MAPK signaling pathway as retrieved from the KEGG database. The red, green, and gray boxes indicate upregulated, downregulated, and nonregulated genes, respectively.

The map of the *Sus scrofa* MAPK signaling pathway in the KEGG database was used as a template, and the location of each of the 38 DEGs in this pathway was confirmed (Figure 3). Many DEGs were located in a key position of this pathway and differentially expressed in the infected piglets versus the control group, such as *MAPK1*, *MAPK8*, *TRAF2*, *GADD45G*, and *BRAF* (Figure 3). Given the same expression trends for the 38 DEGs in the IR group and the IS group, these genes were presented together in a single graph (Figure 3).

**Figure 4:**
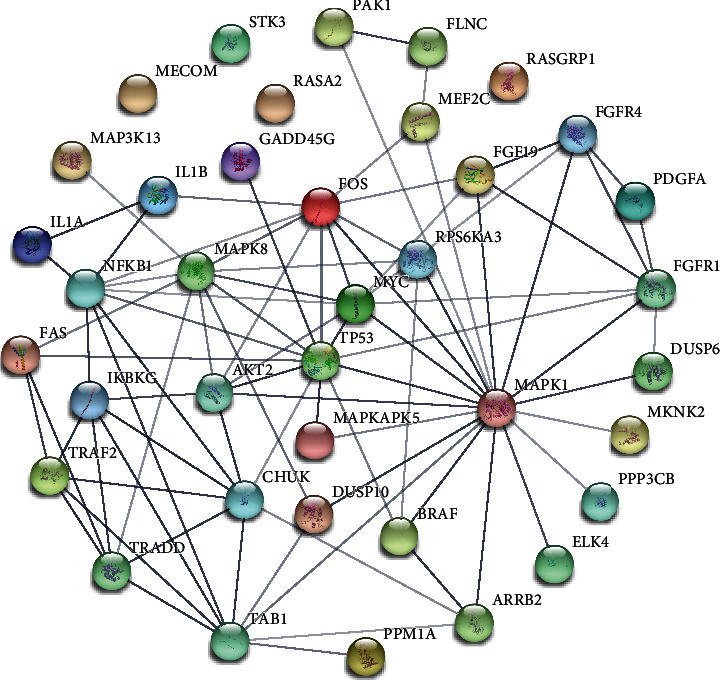
Interactions among 38 DEGs involved in the MAPK signaling pathway. In this PPI network, proteins were represented as nodes and the interactions between two proteins denoted as edges. Active interaction sources: textmining, experiments, databases, coexpression, neighborhood, gene fusion, and cooccurrence. The thickness of the line connecting two genes indicates the strength of the data support.

PPI network analysis revealed that, except for individual genes (*STK3*, *MECOM*, *RASA2*, and *RASGRP1*), most of the genes had strong relationships to each other, with *MAPK1*, *TP53*, *MAPK8*, *MYC*, and *CHUK* lying at the core of the PPI network (Figure 4).

### 3.3. Potential lncRNAs Targeting MAPK Signaling Pathway Genes in the Ileum Tissues of *C. perfringens* Type C-Infected IR and IS Piglets

**Figure 5:**
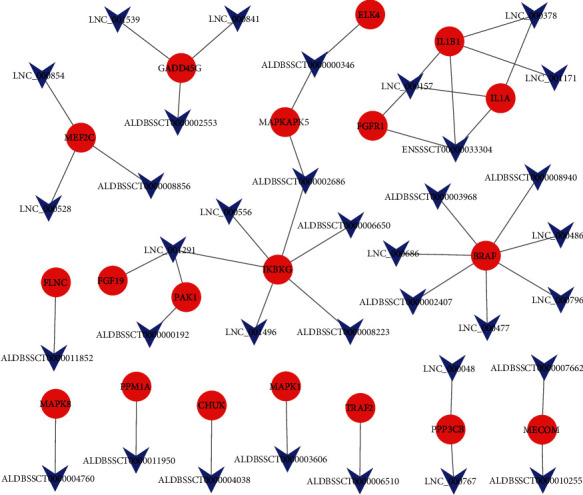
lncRNA-mRNA association network. The interaction network between the 19 DEGs in the MAPK signaling pathway and the 35 lncRNAs targeting them.

We filtered those differentially expressed lncRNAs identified in the ileum tissues between the infected groups and the control group. A total of 19 DEGs from the MAPK signaling pathway were predicted to be targets of 35 DElncRNAs (Table S3). Specifically, we found that ALDBSSCT0000008940, LNC_000486, ALDBSSCT0000002407, LNC_000796, LNC_000477, ALDBSSCT0000003968, and LNC_000686 had common target *BRAF*. *IKBKG* was the common target of ALDBSSCT0000002686, LNC_001291, ALDBSSCT0000008223, LNC_001496, LNC_000556, and ALDBSSCT0000006650. In addition, ALDBSSCT0000004760, ALDBSSCT0000002686, ALDBSSCT0000006510, ALDBSSCT0000006650, and ALDBSSCT0000004038 were shown to be the targets of *MAPK8*, *MAPKAPK5*, *TRAF2*, *IKBKG*, and *CHUK*, respectively. The interactions between mRNAs and lncRNAs are shown in Figure 5.

### 3.4. Quantitative PCR Validation

**Figure 6:**
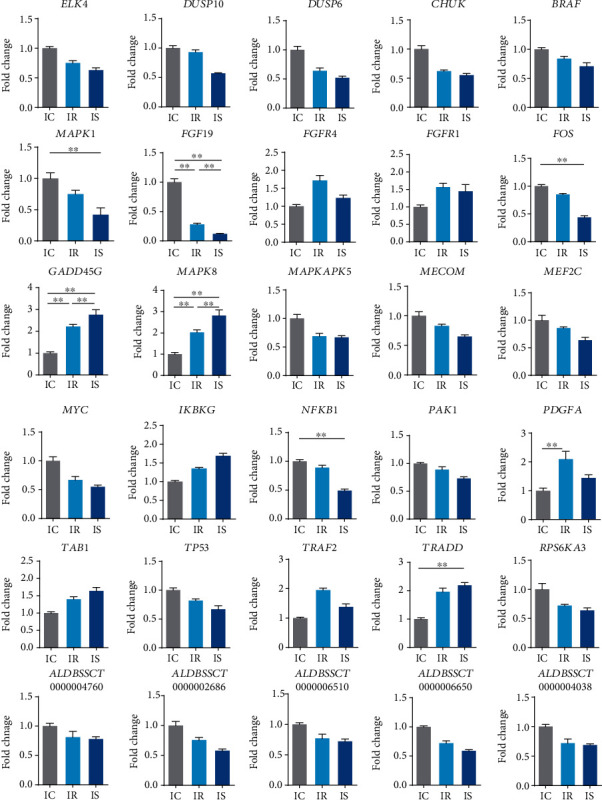
Verification of qRT-PCR for some differentially expressed genes. Examined here are candidate MAPK signaling pathway genes and lncRNAs targeting them that were also differentially expressed in the ileum tissue of *C. perfringens* type C-induced piglets. Bars are the mean ± SD (*n* = 3) and expressed the fold change in gene expression: ^∗^*p* < 0.05 and fold change > 2, ^∗∗^*p* < 0.01 and fold change > 2.

**Figure 7:**
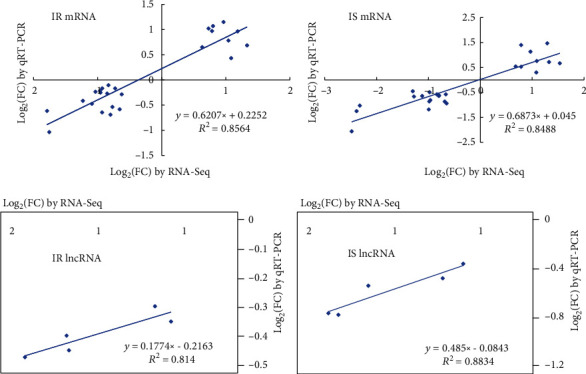
Correlations of log_2_(FC) values between the candidate MAPK signaling pathway genes and the lncRNAs targeting them.

As shown in Figure 6, qRT-PCR results showed that the expression trends of all genes and lncRNAs in the IC group, IR group, and IS group were consistent with the results of RNA-seq. Expression trends were consistent for all transcripts in both analyses, with a coefficient of determination *R*^2^ = 0.8642 for the IR group's mRNAs and *R*^2^ = 0.8488 for the IS group's mRNAs (Figure 7). The expression trends of lncRNA obtained by the above two analytical methods were also consistent, with an *R*^2^ = 0.8140 for the IR group's lncRNAs and *R*^2^ = 0.8834 for the IS group's lncRNAs. These results demonstrated that *C. perfringens* type C infection greatly affected the expression of these MAPK signaling pathway-related genes in ileum tissues of piglets.

## 4. Discussion

*C. perfringens* type C can cause many diseases in animals, such as hemorrhagic enteritis, necrotic enteritis, diarrhea, and even death [28]. Alpha and beta toxins secreted by *C. perfringens* type C can enhance target cell toxicity by activating the MAPK signaling pathway [9, 29]. In rabbit neutrophils, the alpha toxin induces the generation of superoxides through activation of the ERK/MAPK signaling pathway, as reported by Oda et al. [29]. In human lung adenocarcinoma epithelial cell lines, *C. perfringens* phospholipase C (CpPLC) contributes to the production of IL-8 by activating the ERK1/2-nuclear factor kappa B (NF-*κ*B) system and the p38 MAPK system [9]. The beta toxin also induces the phosphorylation of p38 and JNK [10].

This study assessed the differential expression patterns of MAPK signaling pathway genes in the ileum of piglets infected by *C. perfringens* type C using RNA-Seq, qRT-PCR, and bioinformatics. As one of the ancient signal transduction pathways, MAPK is widely used for studying the evolution of many physiological processes [8]. MAPKs are a family of Ser/Thr protein kinases, conserved evolutionarily across all eukaryotic organisms [8], which become activated in response to stimuli to participate in the regulation of a variety of cellular activities, such as gene expression, mitosis, metabolism, motility, survival, apoptosis, and differentiation. The MAPK signaling pathway plays crucial roles in the occurrence and development of inflammatory bowel disease (IBD) [9, 12]. For instance, Waetzig and Schreiber [30] reported that ERK1/ERK2, JNK, and p38MAPK from the MAPK signaling pathway were crucially involved in the intestinal mucosal injury from IBD. Studies have shown that inhibiting the expression of JNK [10, 13] or increasing that of ERK1/2 [31] can reduce intestinal inflammation and epithelial cell apoptosis. Furthermore, JNK and ERK1/2 may be used effectively as therapeutic targets against IBD [13, 31].

There were 14 pathways associated with immune responses, of which the NF-*κ*B signaling pathway [32], toll-like receptor signaling pathway [33], and JAK-STAT signaling pathway [34] are known to be associated with diarrhea caused by *C. perfringens* infection in animals. The above results indicated that *C. perfringens* type C elicited a strong immune response in the ileum tissue of the diarrheal piglets. In addition, 38 DEGs in the MAPK signaling pathway were significantly enriched. Our clustering analysis for gene expression showed two infected groups, namely, IR and IS, clustered together; hence, the established model of piglet diarrhea was successful.

Thirty-eight DEGs screened in this study are located at key positions in the MAPK signaling pathway, thus suggesting this pathway may play a crucial role in piglet diarrhea caused by *C. perfringens* type C. Based on the PPI network of 38 DEGs associated with the MAPK signaling pathway, three hub genes, *MAPK1*, *TP53*, and *MAPK8*, were identified. As an important member of the MAPK system, *ERK* plays multiple roles in regulating inflammatory responses, the production of inflammatory cytokines, and the proliferation and differentiation of epithelial cells. Further, *ERK* can inhibit apoptosis of intestinal epithelial cells [11]. In IBD patients, Waetzig et al. [12] reported that downregulated ERK1/2 expression was capable of inhibiting proliferation and inducing apoptosis of intestinal mucosal cells. In our study, *MAPK1* (*ERK2*) expression was significantly downregulated in the infected piglets, suggesting *MAPK1* could result in intestinal mucosal cell apoptosis in piglets with diarrhea experimentally induced by *C. perfringens* type C.

The protooncogene MYC is one of the transcription factors involved in the occurrence and development of many types of cancer and plays a key role in cell proliferation [35]. Yamaguchi et al. showed that MYC can regulate cell proliferation of intestinal mucosa and participate in the control of cell cycle progression [36]. In colorectal cancer, the upregulated expression of MYC in cancer tissues has been well determined [37]. In our study, the expression of MYC in the IR group and the IS group was significantly lower than that in the control group, which was consistent with the results of intestinal cell apoptosis caused by diarrhea. This indicates that MYC is related to piglet diarrhea caused by *C. perfringens*.

*TP53* is an important transcription factor that participates in stress-induced responses by regulating the expression of genes associated with cell cycle arrest, apoptosis, aging, DNA repair, and metabolic changes [38]. For example, Wang and Friedman [39] found that short-chain fatty acid (SCFA) mixtures can promote apoptosis of colonic epithelial cells by limiting the tumor suppressor protein TP53's expression. In patients with ulcerative colitis, Rosman-Urbach et al. [40] documented that *TP53* gene expression was unstable in the colonic mucosa and low in the serum, indicating that *TP53* is closely related to colonic mucosal inflammation. As an antiapoptotic gene, a decreased expression level of *TP53* should lead to increased activity of the preapoptotic gene caspase 3, thereby initiating an intracellular apoptosis program and causing apoptosis. Our results showed that the expression of *TP53* was lower in the infected groups than that in the control groups, which suggested that the low expression of *TP53* might induce the apoptosis process of the intestinal cells during *C. perfringens* type C infections. Recent work by Girnius and Davis [41] demonstrated that *JNK* can promote the apoptosis of exfoliated epithelial cells. In this study, compared with the IC group, MAPK8 was upregulated in the IS and IR groups, though more so in the former than in the latter, which indicated that JNK might participate in intestinal cell damage caused by *C. perfringens* type C. Continuous activation of *JNK1* during intestinal cell apoptosis can elicit a marked decrease in the expression of *TP53*[39], which is consistent with the changed expression levels of *JNK* and *TP53* in our study here.

The TNFR1-related death domain protein (*TRADD*) is a major adaptor molecule, one crucially involved in the formation of signaling complexes, the induction of apoptosis and necrosis, and the activation of MAPK and NF-*κ*B [42]. Importantly, *TRADD* is engaged in mediating both cell death and proinflammatory signals [43]. In this study, the expression of *TRADD* was significantly upregulated in ileum tissues from infected piglet groups, being most expressed in the IS group. Our findings thus suggest that *TRADD* might promote the apoptosis of intestinal cells, along with having an adverse effect on host defense against infection by *C. perfringens* type C.

*TRAF2*, a key gene in the upper part of the MAPK signaling pathway, participates in regulating the activation of JNK induced by TNF-*α*[44]. TRAF2 may protect the apoptosis of intestinal epithelial cells mediated by TNF-*α*, thereby hindering the inflammation response [45]. Since *TRAF2* shows marked expression in the colon tissue of patients with IBD, there is a potential role for this gene in IBD [46]. Consistent with previous research, in this study, we found that *TRAF2*'s expression increased in the ileum tissues of piglets from infected groups relative to the control group. For infected groups, the expression level of *TRAF2* was higher in the IR than the IS group. These results suggest *TRAF2* may help piglets resist *C. perfringens* infection by regulating their immune and inflammatory responses.

Conserved helix-loop-helix ubiquitous kinase (CHUK) is located downstream of the MAPK signaling pathway, linking the MAPK signaling pathway and the NF-*κ*B signaling pathway. CHUK plays an important role in the negative feedback of NF-*κ*B canonical signaling to limit the activation of inflammatory genes [47]. Rengaraj et al. reported that compared with the control group, the expression of CHUK in chicken necrotic enteritis caused by *C. perfringens* is downregulated [48]. In this study, the *CHUK* in the IR group and the IS group were significantly downregulated compared with the normal group, which is consistent with the above research results.

The growth arrest and DNA damage-inducible gene 45 (*GADD45G*) functions as a stress response protein, having been implicated in various biological processes, such as DNA repair, cell growth, cell differentiation, and apoptosis [49]. GADD45G may participate in the regulation of cell apoptosis by activating the MAPK signaling pathway [50]. Yan et al. found that GADD45G was differentially expressed in the spleen tissue of piglets infected with *C. perfringens*[51]. In our study, *GADD45G* had the highest expression in the IS group, indicating this gene is closely related to intestinal damage and enterocyte death in the ileum of those piglets sensitive to *C. perfringens* type C.

We uncovered 35 lncRNAs, which could somehow participate in regulating the expression of genes located within the MAPK signaling pathway. lncRNA is an important regulator in host defense against bacterial infection diseases [52]. *ALDBSSCT0000006510* could target expression of the *TRAF2* gene, which participates in regulating the activation of JNK induced by TNF-*α*[44]. *ALDBSSCT0000004760* targets the expression of *MAPK8*, a key gene in the MAPK signaling pathway, which was overexpressed in the IR and IS groups compared with the uninfected group. Therefore, *ALBSSSCT0000004760* may participate in the MAPK signaling pathway by regulating the expression of *MAPK8*. All in all, the lncRNAs reported in this study may crucially participate in the development of piglet diarrhea caused by *C. perfringens* type C. However, the specific mechanisms underpinning this regulation still need further investigation.

## 5. Conclusion

In conclusion, this study is the first to screen 38 DEGs involved in the MAPK signaling pathway in piglet ileum tissues infected with *C. perfringens* type C. Most of the DEGs are at key positions of that pathway, of which MAPK1, TP53, MAPK8, MYC, and CHUK belong to the core of the PPI network. In addition, we also identified 35 differentially expressed lncRNAs targeting 19 MAPK signaling pathway genes. Collectively, this work will add to our knowledge of how MAPK signaling pathway genes respond to diarrhea disease in the ilea of piglets infected with *C. perfringens* type C.

## Figures and Tables

**Table 1:** List of 38 DEGs in the ileum of *C. perfringens* type C-infected piglets and involved in the MAPK signaling pathway.

Transcript_id	Gene_id	Gene_name	Gene_location	IR_FPKM	IS_FPKM	IC_FPKM	IR/IS vs. IC
ENSSSCT00000014692	ENSSSCG00000013448	MKNK2	chr2	43.4911	46.414	28.5315	Up
ENSSSCT00000008273	ENSSSCG00000007541	PDGFA	chr3	14.9183	14.7878	8.5737	Up
ENSSSCT00000018048	ENSSSCG00000016578	FLNC	chr18	10.0504	6.34368	2.87348	Up
ENSSSCT00000010506	ENSSSCG00000009585	GADD45G	chr14	7.62666	9.57734	3.91102	Up
ENSSSCT00000035948	ENSSSCG00000000087	TAB1	chr5	16.5275	16.4673	6.57216	Up
ENSSSCT00000011362	ENSSSCG00000010380	MAPK8	chr14	21.0525	21.908	12.6966	Up
ENSSSCT00000006417	ENSSSCG00000005838	TRAF2	chr1	32.5992	41.5492	14.3483	Up
ENSSSCT00000005604	ENSSSCG00000005084	PPM1A	chr1	51.8059	43.5237	34.3071	Up
ENSSSCT00000017223	ENSSSCG00000015815	FGFR1	chr15	3.73792	3.84521	1.81799	Up
ENSSSCT00000036577	ENSSSCG00000014047	FGFR4	chr2	7.0379	7.28958	4.54589	Up
ENSSSCT00000035592	ENSSSCG00000012825	IKBKG	chrX	13.0762	13.1377	6.17629	Up
ENSSSCT00000027163	ENSSSCG00000024182	TRADD	GL896501.1	15.9912	18.1558	9.28888	Up
ENSSSCT00000011849	ENSSSCG00000010831	DUSP10	chr10	7.38813	6.74121	13.1549	Down
ENSSSCT00000006665	ENSSSCG00000006074	STK3	chr4	13.542	12.914	22.2968	Down
ENSSSCT00000019500	ENSSSCG00000017918	ARRB2	chr12	19.7007	16.6525	33.5934	Down
ENSSSCT00000011433	ENSSSCG00000010448	FAS	chr14	11.263	8.86748	19.0862	Down
ENSSSCT00000032467	ENSSSCG00000022284	DUSP6	GL894700.2	34.9735	42.5184	66.7146	Down
ENSSSCT00000008863	ENSSSCG00000008090	IL1A	chr3	0.997492	0.699335	2.9188	Down
ENSSSCT00000028838	ENSSSCG00000025182	ELK4	chr9	29.0925	28.1354	68.2787	Down
ENSSSCT00000010840	ENSSSCG00000009890	MAPKAPK5	chr14	18.5275	17.6704	31.6075	Down
ENSSSCT00000015453	ENSSSCG00000014149	MEF2C	chr2	37.5795	33.6784	73.2693	Down
ENSSSCT00000002650	ENSSSCG00000002383	FOS	chr7	9.18142	9.4395	18.8033	Down
ENSSSCT00000012777	ENSSSCG00000011672	RASA2	chr13	15.7766	14.1345	27.9444	Down
ENSSSCT00000006548	ENSSSCG00000005965	MYC	chr4	28.3306	27.7612	44.6674	Down
ENSSSCT00000019534	ENSSSCG00000017950	TP53	chr12	57.7784	56.1466	89.0769	Down
ENSSSCT00000008861	ENSSSCG00000008088	IL1B1	chr3	0.556422	0.471694	3.69107	Down
ENSSSCT00000014068	ENSSSCG00000012871	FGF19	chr2	3.07843	1.86141	10.3937	Down
ENSSSCT00000005290	ENSSSCG00000004791	RASGRP1	chr1	30.1798	28.8263	51.557	Down
ENSSSCT00000003320	ENSSSCG00000002989	AKT2	chr6	6.50241	8.61669	17.1493	Down
ENSSSCT00000017958	ENSSSCG00000016494	BRAF	chr18	13.8705	13.6419	26.888	Down
ENSSSCT00000011540	ENSSSCG00000010548	CHUK	chr14	14.6343	12.8828	25.4883	Down
ENSSSCT00000012903	ENSSSCG00000011791	MAP3K13	chr13	3.88342	4.13007	6.05921	Down
ENSSSCT00000011042	ENSSSCG00000010081	MAPK1	chr14	26.7106	17.6584	92.3744	Down
ENSSSCT00000027425	ENSSSCG00000011743	MECOM	chr13	3.93771	4.09804	7.1116	Down
ENSSSCT00000035014	ENSSSCG00000014878	PAK1	chr9	24.4613	18.9034	46.5973	Down
ENSSSCT00000035374	ENSSSCG00000010301	PPP3CB	chr14	9.45583	7.1105	17.1076	Down
ENSSSCT00000036028	ENSSSCG00000012163	RPS6KA3	chrX	9.17022	11.2079	19.51	Down
ENSSSCT00000035439	ENSSSCG00000030957	NFKB1	chr8	168.162	48.412	278.469	Down

## Data Availability

All the raw sequencing data have been deposited into an SRA (PRJNA399620) at the NCBI. Other relevant data involved in this study are presented in the Results and Supplementary Materials.

## Supplementary Materials

Supplementary Materials Supplementary materials: Additional file: Table S1: list of differentially expressed genes and immune-related KEGG enrichment pathways in the ileum of piglets infected with *Clostridium perfringens* type C. Table S2: primer sequences for the real-time PCR analysis of genes and lncRNAs associated with MAPK signaling pathway genes. Table S3: list of 35 lncRNAs targeting MAPK signaling pathway genes and differentially expressed in the ileum of *C. perfringens* type C-infected piglets.
